# Understanding the #longCOVID and #longhaulers Conversation on Twitter: Multimethod Study

**DOI:** 10.2196/31259

**Published:** 2022-02-22

**Authors:** Sara Santarossa, Ashley Rapp, Saily Sardinas, Janine Hussein, Alex Ramirez, Andrea E Cassidy-Bushrow, Philip Cheng, Eunice Yu

**Affiliations:** 1 Department of Public Health Sciences Henry Ford Health System Detroit, MI United States; 2 School of Medicine Wayne State University Detroit, MI United States; 3 Sleep Disorders and Research Center Henry Ford Health System Detroit, MI United States; 4 Henry Ford Medical Group Henry Ford Health System Detroit, MI United States

**Keywords:** COVID-19, postacute sequela of COVID-19, PASC, patient-centered care, social media, social network analysis, long term, symptom, Twitter, communication, insight, perception, experience, patient-centered

## Abstract

**Background:**

The scientific community is just beginning to uncover the potential long-term effects of COVID-19, and one way to start gathering information is by examining the present discourse on the topic. The conversation about long COVID-19 on Twitter provides insight into related public perception and personal experiences.

**Objective:**

The aim of this study was to investigate the #longCOVID and #longhaulers conversations on Twitter by examining the combined effects of topic discussion and social network analysis for discovery on long COVID-19.

**Methods:**

A multipronged approach was used to analyze data (N=2500 records from Twitter) about long COVID-19 and from people experiencing long COVID-19. A text analysis was performed by both human coders and Netlytic, a cloud-based text and social networks analyzer. The social network analysis generated Name and Chain networks that showed connections and interactions between Twitter users.

**Results:**

Among the 2010 tweets about long COVID-19 and 490 tweets by COVID-19 long haulers, 30,923 and 7817 unique words were found, respectively. For both conversation types, “#longcovid” and “covid” were the most frequently mentioned words; however, through visually inspecting the data, words relevant to having long COVID-19 (ie, symptoms, fatigue, pain) were more prominent in tweets by COVID-19 long haulers. When discussing long COVID-19, the most prominent frames were “support” (1090/1931, 56.45%) and “research” (435/1931, 22.53%). In COVID-19 long haulers conversations, “symptoms” (297/483, 61.5%) and “building a community” (152/483, 31.5%) were the most prominent frames. The social network analysis revealed that for both tweets about long COVID-19 and tweets by COVID-19 long haulers, networks are highly decentralized, fragmented, and loosely connected.

**Conclusions:**

This study provides a glimpse into the ways long COVID-19 is framed by social network users. Understanding these perspectives may help generate future patient-centered research questions.

## Introduction

The use of social networking sites (SNSs) has grown extensively over the past 10 years, as platforms such as Facebook, Twitter, and Instagram increased in popularity worldwide [1]. Globally, as of January 2021, an estimated 3.6 billion people are using SNSs and use is expected to continue to grow as previously underserved markets gain mobile device usage [2]. SNSs are technologies that support a culture of community sharing, and allow for communication between friends, family members, and strangers spanning geographical, political, or economic borders [3,4]. Typically, SNSs are described as being user-friendly, and include a variety of functions that allow users to communicate with one another while fostering a sense of interpersonal connectedness, as many share their personal stories, struggles, or successes [3]. The reach, engagement, accessibility, collaboration, and advocacy, as well as the research potential of the digital environment can include health messaging, which in turn can influence the attitudes, beliefs, and behaviors of its users [3,5-8].

SARS-CoV-2, and the resulting COVID-19, has contributed to the body of health-related messaging on SNSs, with SNSs serving as a preferred space for communities to connect and share information in real time [9]. A recent scoping review assessing the role of SNSs and COVID-19 suggested six overarching themes in the 81 articles reviewed, including “surveying public attitudes, identifying infodemics, assessing mental health, detecting or predicting COVID-19 cases, analyzing government responses to the pandemic, and evaluating quality of health information in prevention education videos” [10]. Moreover, information about COVID-19 protocols, treatment, personal protective equipment, and allocation of needed resources was disseminated rapidly through platforms such as Twitter [4,10]. Twitter is an SNS that enables users to post short, 280-character messages called “tweets” to their public platform. Data from the first quarter of 2019 show that there were approximately 330 million monthly active Twitter users globally [2]. Recent research has found that throughout COVID-19 (from January 28, 2020, to January 1, 2021), over 132 million tweets from more than 20 million unique users included key words referencing the pandemic [11]. SNSs such as Twitter have fostered a sense of community and togetherness during the social isolation resulting from physical distancing measures and stay-at-home orders [4]. It has now been over 1 year since the onset of the pandemic, and those who were affected by COVID-19 continue to share their experiences on Twitter. In some cases, this includes their experience of being a “COVID-19 long hauler” or having “long COVID-19.”

Describing the 10%-30% of patients diagnosed with COVID-19 that continue to experience symptoms after their infectious period is over [12,13], the terms *COVID-19 long hauler* (ie, the patient) and *long COVID-19* (ie, the disease/symptoms) appear to be common and familiar among both patient-led support groups [14] and the media [15]. COVID-19 long haulers are growing in number, perplexing clinicians and researchers. There is no formal definition or consensus on the terminology for long COVID-19, risk factors for who will be more likely to experience long COVID-19 are still emerging, and there is uncertainty regarding how to alleviate the symptoms of long COVID-19 [16]. The medical and academic communities have described these long-term effects of COVID-19 in several ways. Prolonged symptomatic periods are classified as either “postacute COVID-19,” if the patient is experiencing symptoms for a period greater than 3 weeks, and “chronic COVID-19,” if the patient experiences symptoms for greater than 12 weeks [12]. More recently, the term *postacute sequela* *of COVID-19* has been used to describe symptoms that follow the acute period and can persist for several months [17,18]. As of April 23, 2021, over 144 million people worldwide have been affected by COVID-19 [19] and the unexpectedly high incidence of sequela has become a public health priority.

As the COVID-19 pandemic remains at the forefront of society, and with the debilitating effects of long COVID-19 beginning to surface, research is needed. Leveraging SNSs to understand how this health issue is being framed allows for a unique bottom-up emergent conceptualization. That is, as opposed to traditional media outlets shaping the narrative on a topic, any SNS user is able to control the telling of a story [20,21]. Framing refers to “the process by which people develop a particular conceptualization of an issue or reorient their thinking about an issue” [20]. With the power to share a story from their own perspective through the content they view, share, create, and interact with, SNS users influence how issues are framed, a contrast to the hierarchical gatekeepers of traditional media framing stories and developing headlines. Therefore, the aim of this study was to investigate the #longCOVID and #longhaulers conversations on Twitter by examining the combined effects of topic discussion and social network analysis (SNA) for discovery on long COVID-19. A specific objective included comparing the conversations, understanding the differences and similarities, on Twitter between those discussing long COVID-19 to those narratives created by users identifying as a COVID-19 long hauler.

Specifically, we had the following research questions:

1. What popular/emerging text around #longCOVID and #longhaulers conversations exists on Twitter?
2. What frames did Twitter users employ when discussing long COVID-19?
3. What frames did Twitter users employ when sharing narratives about being a COVID-19 long hauler?
4. What inferences can we draw from the network properties regarding the transmission and adoption of long COVID-19 discourse on Twitter?

## Methods

### Study Design

A multimethod approach was used, which enabled different facets of long COVID-19 on Twitter to be highlighted, leveraged the strengths of two different methods of analysis, and offered several combinatory tactics toward exploration and understanding. In this study, there was an interest in both who is talking with whom and what they are talking about. This emphasizes the interest in both the network of social connections and the nature of the tie that underpins these connections [22]. Data collection for Twitter was performed using the Netlytic program [23], followed by text and social network analyses.

### Ethical Considerations

The Netlytic program uses application programming interfaces (APIs) to collect publicly accessible posts from Twitter [23]; therefore, the activities described do not meet the definition of human subjects research and did not require institutional review board review.

### Netlytic Analysis

Using the Netlytic program [23], an open-source software, all publicly accessible, tagged media with the #longCOVID AND/OR #longhaulers hashtag on Twitter were downloaded (ie, when the tweet was tagged, not necessarily when it was posted). The download, initiated by the lead author (SS), was specified to remove all non-English tweets and retweets, and occurred on February 23, 2021 (data were pulled until the maximum data set allowed by the software was built; N=2500). A tweet is a post made on Twitter and the term *record* will be used interchangeably throughout the article. The data set consisted of records retrieved from February 18, 2021, to February 23, 2021. Specifically, for this study, Netlytic [23] was used to identify popular topics in the #longCOVID AND/OR #longhaulers data set, as measured by word frequency. Furthermore, Netlytic [23] was used to perform a network analysis around #longCOVID AND/OR #longhaulers, including both a *Name* network (ie, who mentions whom) and a *Chain* network (ie, who replies to whom).

The Twitter records (N=2500) were downloaded as an output file (in Excel) for further analysis. The Netlytic program [23] produced an output file (in Excel) that recorded the link to the tweet, including the publication date, number of times the tweet was liked, and number of times the tweet was retweeted. The output file also included information about the author of the tweet, including their Twitter handle, link to their profile image, frequency counts on the author’s total number of tweets (including retweets), total number of followers, and total number of users the account is following.

The data-cleaning process as well as the multimethod approach utilized (ie, text and network analyses) are discussed in further detail below.

### Data Cleaning

Four independent coders were each provided an equal portion (n=625 records) of the output file (N=2500 records). The terms “records” and “tweets” are used synonymously. To address the research questions of the study, two distinct groups of records were created: (1) *tweets about long COVID-19* and (2) *tweets by COVID-19 long haulers*. To specifically delineate these two groupings of records, each coder was instructed to read and identify the record as to whether it had been constructed/posted by a self-identified COVID-19 long hauler. Those records constructed/posted by a self-identified COVID-19 long hauler were labeled as *tweets by COVID-19 long haulers*, with remaining records falling into the *tweets about long COVID-19* data set. To accomplish this delineation, coders were trained to review the record holistically and to specifically look for personal pronouns (eg, I, my). The holistic approach and personal pronouns were used to identify *tweets by COVID-19 long haulers* because it appeared that these records were of self-reflection and/or a Twitter user sharing their narrative about being a COVID-19 long hauler. In addition, coders were required to read all records thoughtfully and with an objective lens.

During this data-cleaning process, which was considered a time of familiarization with the data, the coders were also instructed to record meaningful units of text or codes that they felt were emerging from the records. As a holistic approach was utilized, while analyzing tweets, coders were instructed to view any emojis used as part of the record. The final data corpus consisted of 2010 *tweets about long COVID-19* and 490 *tweets by COVID-19 long haulers*. These data sets were considered separately in the text and network analyses.

### Text Analysis

#### Computer Coding to Identify Popular/Emerging Text

The final data corpus was uploaded back into Netlytic [23] and the Keyword Extractor tool was used. This computer-automated coding first removes all common words such as “of,” “will,” and “to” from a list of stop words in the English language. It then counts the number of records where a unique word appears, thus identifying popular topics in the data set, as measured by keyword frequency. Although Netlytic [23], as a qualitative data collection tool, provides several advantages (eg, objective, ability to analyze a large data set), it can miss the nuances or specifics within the data set. Therefore, human coding was also performed to further contextualize the content.

#### Human Coding to Identify Emerging Frames

Although there are no uniform measurement standards on how to identify/define a frame in communications, the most persuasive studies use a four-step method [20], which was utilized in this study. The first step requires that an issue or event is identified [20], which in this study is that of long COVID-19 and those suffering from the aftermath. The second step involves isolating a specific attitude [20], which in this study was the overall attitudes toward long COVID-19. In the third step, an initial set of frames is identified inductively to create a coding scheme [20]. In this study, this third step was developed after the familiarization period, and then the four independent coders discussed possible codes and themes within the data sets. Separate codebooks for *tweets about long COVID-19* and *tweets by COVID-19 long haulers* were mutually agreed upon. All coding of themes (12 themes for *tweets about long COVID-19*, 13 themes for *tweets by COVID-19 long haulers*) was completed independently, and records could have been coded into different themes (thus potentially overlapping). Each coder revisited their originally assigned data set (n=625); however, at this stage, records were organized by *tweets about long COVID-19* and *tweets by COVID-19 long haulers*. For trustworthiness and rigor, the lead author (SS) also coded ~10% of the other three coders’ data (n=63 records/coder for a total of 189). Similar to previous studies [24,25], 30% of the data corpus was selected by the authors as a feasible and manageable strategy that would still capture sufficient variation in responses [26]. It has been suggested that multiple coding can be a valuable process for interrater reliability and refining interpretations or coding frameworks, but multiple coding of entire data sets is not recommended [26].

The fourth and final step involves using the coding scheme to complete a content analysis [20]. Thus, once a complete understanding of the themes was attained, the four coders engaged in axial coding as a group, which consisted of regrouping or reducing themes into frames based on similar dimensions [27]. In total, four prominent frames were identified for *tweets about long COVID-19* (“research,” “support,” “medical care,” and “political”) and four prominent frames were identified for *tweets by COVID-19 long-haulers* (“advocacy,” “symptoms,” “building a community,” and “medical care”). In *tweets about long COVID-19*, 79 records (3.9% of the sample) did not fit into any of the themes and subsequent frames. In *tweets by COVID-19 long haulers*, 7 records (1.4% of the sample) did not fit into any of the themes and subsequent frames. For both data sets, these “outlier” records consisted of tweets comprised of only hashtags as well as tweets that were too out of context to interpret confidently and objectively (eg, tweet comprised of single words or emojis, replies to other threads). Thus, the remaining 1931 and 483 records for *tweets about long COVID-19* and *tweets by COVID-19 long haulers*, respectively, were included in axial coding and overall frames (see the Manual Coding to Identify Emerging Frames subsection in the Results).

Lastly, intraclass correlations were computed using IBM SPSS Statistics (version 25) to determine the interrater reliability of the frames using a two-way random, single-rater, average measures model [28]. Minimum acceptable levels of agreement (0.40-0.75) [29] were observed for all frames.

### Network Analysis

SNA can help in understanding how and why COVID-19 long haulers in a network are connected; how they seek each other out; and how their connections, configurations, and interaction patterns support information and knowledge sharing. Thus, a network perspective can provide several novel ways that long COVID-19 can be represented and addressed, guide efforts in medical care, and aid in designing future research questions. To explore the social connections underlying the online conversations being examined, the final data corpus was uploaded back into Netlytic and the Network Analysis tool was used [23].

Both *Name* and *Chain* networks were generated for both *tweets about long COVID-19* and *tweets by COVID-19 long haulers.* The *Name* network was used to show connections between online participants based on direct interactions such as replies or based on indirect interactions such as mentions or retweets [23]. A person’s mentions capture a sense of acknowledgment and their retweets capture instances of endorsements. The *Chain* network connects participants based on their posting behavior and usually includes only direct interactions [23], meaning a tweet that includes a username. Both *Name* and *Chain* networks have been validated and applied in different contexts, including Twitter communities [30,31].

## Results

### Tweet Characteristics

Table 1 summarizes the overall descriptive statistics of the Netlytic output file for both *tweets about long COVID-19* and *tweets by COVID-19 long haulers*. On average, *tweets by COVID-19 long haulers* are liked more (ie, favorite count) than *tweets about long COVID-19*. Conversely, *tweets about long COVID-19* are retweeted more, on average, than *tweets by COVID-19 long haulers*.

**Table 1:** Descriptive statistics of Twitter records (ie, tweets) from a one-time Netlytic data pull in February of 2021.

Characteristic	*Tweets about long COVID-19* (n=2010)			*Tweets by COVID-19 long haulers* (n=490)	
	Range	Mean (SD)	Range		Mean (SD)
Favorite count^a^	0-1067	10 (52.0)	0-4614		17.2 (209.1)
Retweet count^b^	0-498	3.6 (23.1)	0-1039		3.3 (47.2)
User statuses count^c^	6-1.69×10^6^	3.00×10^4^ (7.48×10^4^)	5 (1.69×10^6^)		4.56× 10^4^ (2.16×10^5^)
User friends count^d^	0-3.80×10^5^	2.33×10^3^ (1.22×10^4^)	0 (3.07×10^4^)		1.65×10^3^ (3.03×10^3^)
User followers count^e^	0-2.57×10^6^	7.30×10^3^ (6.62×10^4^)	0 (4.48×10^5^)		9.54×10^3^ (5.69×10^4^)

^a^Number of times the tweet has been liked.

^b^Number of times the tweet has been retweeted.

^c^Number of tweets (including retweets) issued by the user.

^d^Number of users the account is following.

^e^Number of followers the account currently has.

### Computer Coding to Identify Popular/Emerging Text

**Figure 1:**
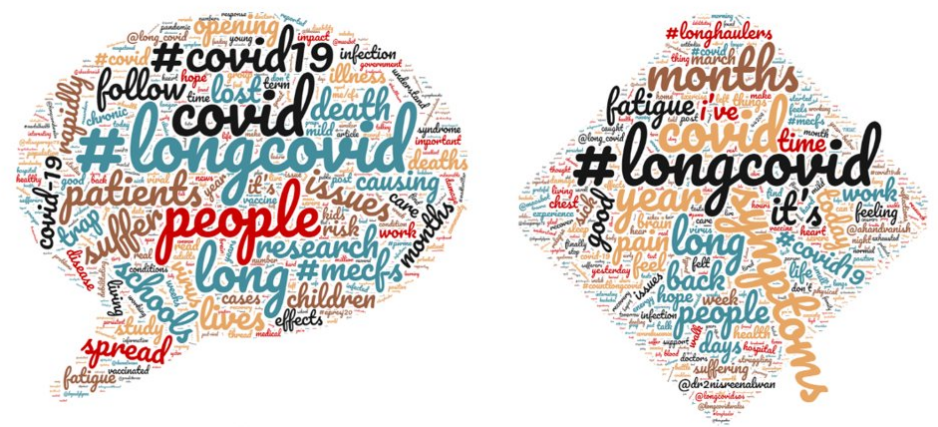
Word cloud of tweets about long COVID-19 (left) and tweets by COVID-19 long haulers (right) based on number of instances from a one-time Netlytic data pull in February of 2021.

Among the 2010 *tweets about long COVID-19*, 30,923 unique words were found. Among the 490 *tweets by COVID-19 long haulers*, 7817 unique words were found. Figure 1 provides an exploration of frequently tweeted words (a larger, more pronounced word reflects a greater frequency), allowing for a possible text-mining approach that can be applied to our data set.

For both *tweets about long COVID-19* and *tweets by COVID-19 long haulers* “#longcovid” and “covid” were the most frequently mentioned words. With *tweets about long COVID-19,* “#longcovid” was mentioned 1951 times (n=1913 records, 95.2%) with “covid” mentioned 479 times (n=429 records, 21.3%). In the *tweets by COVID-19 long haulers*, “#longcovid” was mentioned 478 times (n=470 records, 95.9%) with “covid” mentioned 96 times (n=83 records, 16.9%). Of interest in the current study were the nuanced differences between the *tweets about long COVID-19* and *tweets by COVID-19 long haulers*. By visually inspecting the data, it appears that words relevant to having long COVID-19 (ie, symptoms, fatigue, pain) are more prominent in the *tweets by COVID-19 long haulers*. Further comparisons in the most frequently used words for both *tweets about long COVID-19* and *tweets by COVID-19 long haulers* can be found in Table 2.

**Table 2:** Top 30 words in tweets about long COVID-19 and tweets by COVID-19 long haulers conversations on Twitter from a one-time Netlytic data pull in February of 2021.

Term			Number of records		Number of instances
***Tweets about long COVID-19* (n=2010 tweets; 30,923 unique words)**					
	#longcovid	1913		1951	
	covid	429		479	
	people	308		344	
	#covid19	272		277	
	long	253		279	
	symptoms	197		209	
	patients	146		157	
	issues	139		140	
	suffer	135		136	
	lives	132		134	
	schools	131		131	
	death	131		132	
	thousands	129		133	
	follow	126		128	
	#mecfs	123		130	
	@borisjohnson	121		126	
	health	117		126	
	spread	116		116	
	#longhaulers	116		116	
	lost	115		115	
	families	114		114	
	research	114		132	
	dangerous	111		111	
	respiratory	110		110	
	causing	106		106	
	opening	106		106	
	suffering	105		107	
	@parents_utd	105		105	
***Tweets by COVID-19 long haulers* (n=490 tweets; 7817 unique words)**					
	#longcovid	470		478	
	covid	83		96	
	symptoms	64		69	
	months	61		64	
	year	59		64	
	long	54		59	
	it’s	43		51	
	people	41		45	
	back	38		41	
	i’ve	37		37	
	fatigue	35		40	
	pain	35		40	
	good	35		36	
	#covid19	34		34	
	work	32		34	
	time	31		34	
	today	31		35	
	feel	27		28	
	days	27		30	
	#longhaulers	24		25	
	hope	24		25	
	March	23		23	
	sick	22		25	
	life	21		23	
	week	20		23	
	brain	20		21	
	feeling	20		21	
	suffering	19		20	

### Manual Coding to Identify Emerging Frames

#### Overview

The results are presented in multiple formats to demonstrate the similarities and differences within the frames, and between *tweets about long COVID-19* and *tweets by COVID-19 long haulers*. Examples and the prevalence of each frame are provided in Table 3.

**Table 3:** Prevalence and examples of emerging frames identified by manual coding in *tweets about long COVID-19* and *tweets by COVID-19 long haulers* conversations on Twitter from a one-time Netlytic data pull in February of 2021.

Frame		Themes	Prevalence, n (%)	Examples^a^
***Tweets about long COVID-19* (n=1931)**				
	Support	resources/ information, advocacy, financial, well wishes, skepticism	1090 (56.4)	“The weekly @LongCOVIDGuide newsletter is your guide to the latest news and research about Long Covid! #LongCovid” “When Does COVID-19 Become A Disability? ‘Long-Haulers’ Push for Answers, and Benefits #Pharma #Rx #COVID19 #LongHaulers” “#LongCovid is forcing thousands of people --likely millions in US-- to leave their jobs and stop working. The health impacts from Covid may be lifelong and disabling many people. The impact this will have on our long-term economy is MASSIVE. Plus the massive health care costs.” “Thanks to journalists who continue to investigate &amp; share important articles. Thanks to #LongHaulers who share their stories. Our community knows it is not easy but it can be powerful.” “So sorry you are having to scale back & modify things. As discouraging as it is, it looks like you are doing what you need to...to preserve function and get through your day. Big air hugs to you. Will continue to wish you well as you navigate living w #LongCovid [prayer hands emoji]” “Do you remember the 34 pandemics we had in the 1970s/80s/90s and 2000s, before the 2020 Covid pandemic - all worse? Do you remember the 34 previous lockdowns? No, me neither. Maybe I’ve got brain fog as a result of unknowingly contracting #LongCovid.”
	Research	research needed, ongoing research/research findings, research funding, research on self/home or alternative remedies	435 (22.5)	“Any experts /trial to see if monoclonal antibodies may help in viral persistence / #LongCovid?” “We have open sourced our #LongCOVID survey and it’s available to use (with citation) in 9 languages” “The hypothesis that viral persistence of #SARSCOV2 in the body causes an ongoing immune response in patients with #longcovid is gaining ground. From Spain, this rationale written by our patient-led research team: https://t.co/o26apP0zOa #MedTwitter #Covid19 #covidpersistente” “Or, expand your study - suspect given the large numbers of #LongCOVID patients without a history of positive tests, esp antibody tests (incl those who tested + for infection) that they represent an important immunological phenotype to study” “Some interesting data regarding gender and Covid-19. Back in April I mentioned men are far more likely to die of Covid-19 than women. This is still true to this day but also very interesting is that women are significantly more likely to get #LongCovid than men. [confused face emoji]” “The 2021 RFA for our Ramsay Grant Program, which funds pilot studies into #ME/CFS + #LongCovid, is now open! For information on types of grants, previously funded research, how to apply, + more please visit https://t.co/PLHJbr4uUt” “#LongCovid - @groundology - UK - Grounding/Earthing - solution to get out of Covid ill-health. Medical drugs will not resolve ALL. Ancient remedy modernised. Read research first”
	Medical care	treatment, links to chronic disease	396 (20.2)	“Geez, we’re up to 3 #LongCOVID clinics in Vancouver now. I hope Ohio gets with the program.” “Disturbing news: #Covid19/ #Longcovid, maybe an early way for some towards Alzheimer disease. Biochemical pathways activated by #SarsCoV2 infection.” “I’ve spent the last 11 years waiting for a cure for #mecfs but nothing yet I'm afraid. I think #LongCovid will actually help because so many more people are unwell and we can join forces to get this looked into!” “I can not help but wonder, if the medical community had taken Chronic Fatigue Syndrome (#CFS/#MECFS/#CFIDS/#SEID) more seriously, instead of trivializing the illness, could they have been prepared for these perplexing #LongCovid abnormalities that emulate #CFS? #SARSCoV2 #COVID19”
	Political	politicians/ parties/plans	311 (16.1)	“what is the government doing for #LongCovid they never seem to answer” “Really want to see questions and discussion on the BIG issue of #LongCovid now in these government broadcasts.” “#LongCOVID: The disease UKGOV barely acknowledges, doesn’t care enough to mitigate against, and refuses to name. [angry face emoji]” “Well said @GwynneMP - we need access to clinics and therapeutics for everyone with #LongCovid Thank you for reminding the PM about this issue!”
***Tweets by COVID-19 long haulers* (n=483)**				
	Symptoms	mental health, physical health, comparing health time points	297 (61.5)	“I learned around month 5 not to self cheer so much after feeling ‘a little better’ one day. Long haul was such an appropriate term! Mind game... do you still tell anyone when a symptom improved? I’ve been on both sides of that answer, just as #LongCovid said ‘nah, im still here’” “Day 320 of living with #LongCovid and the relapse continues. My body and brain were so exhausted today I struggled to get out of bed all day. Fatigue has reduced around 6pm but very aware that energy could evaporate very quickly, so still focused on rest” “Yup. In the beginning, I got sick (like a bronchitis) once a month. Now I get better once a month. My asthmatic lungs are worse than ever. EVERYTHING is too much. It's been 1 year. I do all the things they say and keep getting worse. #longcovid”
	Building a community	pride/ accomplishment, well wishes, advice, searching for support	152 (31.5)	“Recommendations for a winter running jacket? Now doing intermittent jog/walks. Jog for 1 count of 8, walk for 3-5x8. This is how a dancer builds up reconditioning ;) [dancer emoji] It’s a HUGE improvement. I’m hoping in 4-8weeks I’ll be able to go on a full run. #LongCovid #LongCovidRecovery” “TaiChi, Wild swimming, meditation, mindfullness have all been in my #LongCovid tool kit along with all the conventional treatment and rehab... https://t.co/0uu9HVgxfH” “Anyone have any good tips/tricks/home remedies for the #longcovid GI flare up (nausea, vomiting, gastritis-type pain, all the GERD stuff)? I have a doctors appt in 10 days ish so more looking for recommendations for teas, supplements etc than meds” “Finding an online #longcovid FB group in early May last year was a godsend. To just know others were going through the same thing was weirdly reassuring, despite the snakes and ladders nature of this beast. Solidarity is so powerful.”
	Advocacy	awareness, employment, disability	106 (22.0)	“Please read. This is so true. We need research. We need help. We are #longhaulers #COVID19” “It’s really hard to hear ‘it’s not your fault, you’re doing everything right, but you’re still going to lose your job’ #COVID19 #LongCovid #longhaulers” “According to NHS, in January, 25% of hospital admissions were for people under 55. And ONS found that 10% of CV19 sufferers will go on to develop debilitating #longcovid at cost to individual, families and economy. As a 48yo with #longcovid I can confirm this thing is a shit.”
	Medical care	access to care, experience with clinicians/health care, COVID-19 vaccine	79 (16.4)	“I am rapidly approaching a year now with no let up of #longcovid symptoms. No Long Covid clinic in Sunderland so no programmes of support being offered. But things have improved incrementally. Vit D helps” “Even doctors also not believe my symptoms then how my company HR? #LongCovid” “I had a very rough time of it; now back to the previous #LongCovid symptoms. Vaccine hasn’t had any positive effect, at least not yet. It’s been 13 days...”

^a^Example tweets have been paraphrased/slightly modified so they are not easily searchable for user identification.

#### Tweets About Long COVID-19

##### Main Frames

Analysis of the *tweets about long COVID-19* revealed that the most discussed frames were “support” and “research,” followed by “medical care” and “political” (Table 3). Frames are discussed in further detail below.

##### Support

Records in this frame contained messaging indicating some form of support for long COVID-19. For almost all the records coded in this frame, the support was viewed with a positive connotation, including mention of support groups, petitions, the need for long COVID-19 to be recognized as a disease and serious health problem, and supportive messaging and/or advice.

How many Long Haulers are there? They matter - everyone matters. Never forget them; [or] stop supporting them. Let’s use an orange heart to support them. Never forget the over 500,000 Americans who lost their lives - many could have been prevented #LongHaulers #MaskUp

There were 29 records that were against supporting long COVID-19 and discussed conspiracy, used cynicism, or criticized long COVID-19 and the long haulers: “#LongCovid is an absolute myth. Even if it were real - there is no threat of death from it. Therefore no excuse for more lockdowns.”

##### Research

This frame included records that focused on all aspects of research, including funding available, recruitment of ongoing research, and findings, with links to publications. Interestingly, Twitter users were posing research questions or calls to action, such as “Is anyone studying - or even publicly questioning - whether and how environmental factors may be influencing or contributing to people’s experiences of #LongCovid?” In addition, this frame also included records mentioning home remedies or alternative medicine being researched for long COVID-19, such as “The #longcovid snake oil treatments and medicines popular in the patient-led covid groups are horrifying and profoundly sad. There must be light shed here.”

##### Medical Care

This frame discussed the current views on treatment and/or the need for treatment options, which encompassed clinical services available, diagnostics, as well as denial of care: “#LongCovid clinics out there requiring a positive PCR/serology, think long and hard about what you’re doing.” In addition, this frame included records that mentioned the COVID-19 vaccine as a possible treatment method: “Is there any evidence that vaccine prevents #LongCovid or covid lung? Are we sure it prevents other long-term issues from vaccinated infection?” Lastly, this frame delved into the narrative around how long COVID-19 is related to or associated with diseases or the development of comorbidities.

Encouraged by coordination of the #Covid-19 research points to the role of post viral inflammation from SARS-CoV-2, leading researchers to compare Covid-19 to other chronic diseases such as #MEcfs #pwme #myalgicE #millionsmissing #longhauler #LongCovid #COVID19.

##### Political

Records in this frame focused on content that was politically driven, mentioning political parties, policy decisions, or specific politicians.

#COVID19 is not like the flu @BorisJohnson. It leaves 10% of people with long-term morbidity - did you forget? If we don't control it this will have a significant impact on society and the economy #LongCovid.

##### Overlap

All *tweets about long COVID-19* frames experienced some overlap, with 702 (36.3%) records having been coded in multiple frames. Frames that overlapped the most were “support” with “medical care” in 289 records, or 15.0% of the entire data set, followed by “research” with “medical care” in 233 records, or 12.1% of the entire data set. Frames that overlapped the least were “research” with “political” in 4 records, or 0.2% of the entire data set.

#### Tweets by COVID-19 Long Haulers

##### Main Frames

Most of the *tweets by COVID-19 long haulers* focused on “symptoms” the individuals were experiencing and “building a community,” followed by “advocacy” and “medical care” (Table 3). Frames are discussed in further detail below.

##### Symptoms

Records in this frame made mention of mental and/or physical health status, linking their experiences to other medical conditions, and COVID-19 long haulers making comparisons to their life before and after having COVID-19.

Day 321 of living with #LongCovid. After yesterday’s extreme fatigue where in the day I often didn’t have the energy to move my arms. The night was the other extreme, insomnia so bad that I couldn’t sleep all night as if someone had put me on an IV drip of caffeine. Bonkers 
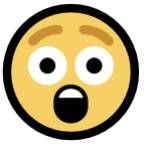

##### Building a Community

This frame emphasized COVID-19 long haulers sharing their stories of accomplishments and failures, providing supportive and/or empathetic messages, offering advice and/or treatment modalities, as well as those seeking to gain a support network of others experiencing long COVID-19: “Feeling a little blue because I’m suffering from #longcovid... Anyone out there going through the same? Would love to chat...
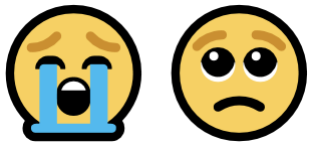
.”

##### Advocacy

This frame discussed the need for long COVID-19 to be recognized as a disease and as a disability: “Right now I write #LongCovid on my dashboard to park in the handicapped spot 
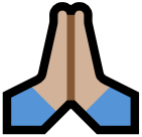
 can’t wait to have a ribbon for my car instead.” Records in this frame also consisted of messages around the negative impact of being a COVID-19 long hauler on employment, a need for resources, petitions, crowdsource funding, and the importance of research.

##### Medical Care

Records in this frame describe the perspective of someone with long-haul COVID-19 on access to care, and experience with clinical services, providers, and/or treatments.

The medical community is still in denial of #LongCovid/#PACS, so how could the public be understanding? Doctors are blaming my symptoms on anxiety, supplements...anything but COVID. Infectious disease doc still using lack of positive test against me (both for Lyme and COVID).

In addition, records that discussed the pros and cons of getting the COVID-19 vaccine as a treatment for or protection against long COVID-19 were also grouped under this frame.

I hope #LongCovid suffered see this. From what I’m reading, sufferers are getting dreadfully hammered by the vaccine. Don’t forget, we have an autoimmune problem that cause serious trauma brain cytokine storms. Get the vac if you want, but don’t feel forced or coerced.

##### Overlap

All of the *tweets by COVID-19 long haulers* frames experienced some overlap, with 100 (20.70%) records having been coded in multiple frames. Frames that overlapped the most were “symptoms” with “building a community” in 38 records, or 7.87% of the entire data set, and “symptoms” with “advocacy” in 27 records, or 5.59% of the entire data set. Frames that overlapped the least were “building a community” with “medical care” in 4 records, or 0.83% of the entire data set.

### Social Network Analysis

Table 4 highlights the findings of the network analysis generated by Netlytic [23]. Two Twitter accounts will be connected in the *Name* network if one replies to or mentions another in their message. The *Chain* network is a subset of the *Nam*e network because it only connects people if one replied to another.

**Table 4:** Social network analysis of tweets about long COVID-19 and tweets by COVID-19 long haulers conversations on Twitter from a one-time Netlytic data pull in February of 2021.

Characteristic	*Tweets about long COVID-19*, n		*Tweets by COVID-19 long haulers*, n	
	Name network^a^	Chain network^b^	Name network	Chain network
Network actors with ties^c^	648	396	156	121
Ties (including self-loops)	2923	1653	478	389
Names found^d^	2406	N/A^e^	608	N/A

^a^Who mentions whom: a communication network built from mining personal names in the messages.

^b^Who replies to whom: a communication network built based on participants’ posting behavior.

^c^Network actors are members connected together based on some common form of interaction (“ties”) [23].

^d^Number of unique personal names that Netlytic found in this data set.

^e^N/A: not applicable.

**Figure 2:**
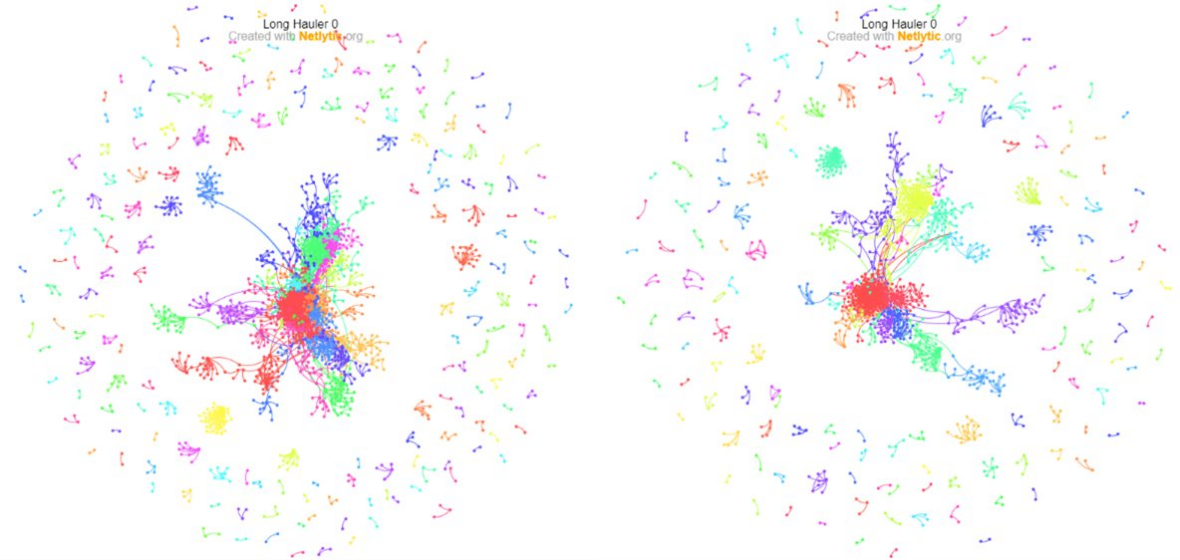
*Name* (left) and *Chain* (right) networks for tweets about long COVID-19 conversations on Twitter from a one-time Netlytic data pull in February of 2021, presented using a Dr L layout [30].

**Figure 3:**
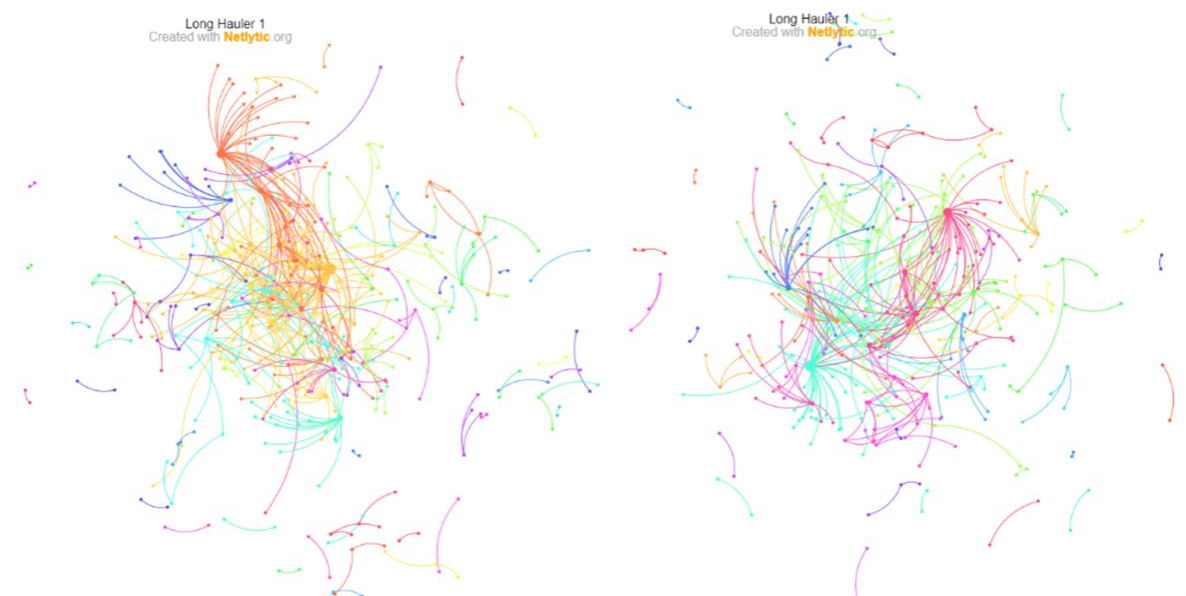
*Name* (left) and *Chain* (right) networks for tweets by COVID-19 long haulers based on conversations on Twitter from a one-time Netlytic data pull in February of 2021, presented using a Fruchterman-Reingold layout [31].

Figure 2 and Figure 3 show the *Name* and *Chain* networks built from the #longcovid #longhaulers data set, split by *tweets about long COVID-19* and *tweets by COVID-19 long haulers,* constructed using a Dr L layout [32] and a Fruchterman-Reingold layout [33], respectively, which are force-directed graph–drawing algorithms effective for large networks (<1000 nodes). The node colors are assigned automatically (based on the “Fast Greedy” community detection algorithm) [34]. Each color represents a group of nodes more likely to be connected to each other than with the rest of the network. Based on visual inspection of the networks, the *Chain* network has fewer nodes. This is somewhat expected since it only represents direct replies between Twitter users. The clustering and network fragmentation aspects at the macrolevel are discussed in the following section.

### Macrolevel SNA Measures

Macrolevel SNA measures that are found to be useful when analyzing and comparing different social networks include density, reciprocity, centralization, and modularity [35]. Table 5 depicts Netlytic’s five measured network properties, which describe network characteristics such as how individuals interact with each other, how information flows, and whether there are distinct voices and groups within the network [23].

The diameter property provides a measure of network size. The diameter property was different between the *Name* network and the *Chain* network in *tweets about long COVID-19.* For the *Name* network in *tweets about long COVID-19*, it may take up to 100 connections for information to travel from one side of the network to the other. Smaller values for the diameter indicate a more highly connected network, which is true for the *Chain* network in *tweets about long COVID-19* as well as the *Name* and *Chain* networks in *tweets by COVID-19 long haulers.* The density property is complementary to diameter, as both assess the speed of information flow, with density helping to illustrate how close participants are within a network. As the density property is closer to zero for both network types, in the *tweets about long COVID-19* and *tweets by COVID-19 long haulers,* this suggests there is not a close-knit community and participants are not talking with others.

The conversations for both *tweets about long COVID-19* and *tweets by COVID-19 long haulers* appear to be one-sided, with little back-and-forth conversation, as indicated by the low reciprocity values for all networks. Moreover, the conversations for both *tweets about long COVID-19* and *tweets by COVID-19 long haulers* show decentralization (ie, closer to 0). This low centrality score suggests that the networks contain a number of influential participants, but there is not a single opinion leader (eg, informed, respected, and well-connected individuals) controlling the conversation [8]; there was a free flow of information between the users. Finally, the last property, modularity, is dependent on clusters within the network. A cluster is a group of densely connected nodes that are more likely to communicate with each other than to nodes outside of the cluster. The higher value of modularity (>0.5) both for *tweets about long COVID-19* and *tweets by COVID-19 long haulers* in all networks indicates clear divisions between communities, and thus the clusters do not overlap. The network does not consist of a core group of nodes and consists of different conversations as well as communities with weak overlap.

**Table 5:** Detailed network property descriptions and results for Twitter social network analysis in *tweets about long COVID-19* and *tweets by COVID-19 long haulers* conversations on Twitter from a one-time Netlytic data pull in February of 2021.

Network properties	Description^a^	Tweets about long COVID-19		Tweets by COVID-19 long haulers	
		Name network	Chain network	Name network	Chain network
Diameter	Calculates the longest distance between two network participants	100	9	5	5
Density	A proportion of existing ties to the total number of possible ties in a network	0.000588	0.000828	0.002362	0.002778
Reciprocity	The number of reciprocal ties (two-way conversations) compared to the total number of ties	0.021690	0.022550	0.031110	0.027400
Centralization	How freely information flows within a network	0.020630	0.030920	0.049470	0.058320
Modularity	Whether the clusters found indicate distinct communities in a network	0.819600	0.850600	0.802400	0.805100

^a^Descriptions are based on Mitchell et al [36] and Gruzd et al [22].

## Discussion

### Principal Findings

The objective of this study was to use a multimethod approach to compare the conversations on Twitter between those discussing long COVID-19 to the narratives created by users identifying as COVID-19 long haulers. Selected findings reflect that many of the users who tag their tweets with #longCOVID and #longhaulers seem to be doing so to highlight the outcomes and implications of the COVID-19 pandemic, similar to previous Twitter studies on COVID-19 [4,10]. In addition, compared to *tweets about long COVID-19, tweets by COVID-19 long haulers* appear to be more frequently mentioning words relevant to having long COVID-19. Manual coding identified that the most prominent frames employed when discussing long COVID-19 were “support” and “research.” Conversely, “symptoms” and “building a community” were the frames most prominent in conversations by COVID-19 long haulers. Lastly, SNA provided insight into network typologies, and inferences were drawn regarding the transmission and adoption of long COVID-19 discourse on Twitter. For both *tweets about long COVID-19* and *tweets by COVID-19 long haulers*, networks appear highly decentralized, fragmented, and loosely connected. Overall, the results provide insight into how long COVID-19 is being framed from the perspective of SNS users, and allows for those users to decide what and how topics and issues are being presented to the broader health community.

Regarding long COVID-19, this study has important clinical and academic relevance, and can act to inform care and research moving forward. Our findings can influence clinical practice guidelines for long COVID-19, playing an important role in ensuring the delivery of high-quality health care. As clinical practice guidelines provide recommendations for how best to treat a typical patient with a given condition [37], utilizing Twitter conversations can provide broad perspectives and experiences from various stakeholders. Previous literature has indicated that engaging stakeholders with legitimate interests in the development of clinical practice guidelines can improve quality and utility [38]. Long COVID-19 is currently understood and defined by patient-reported symptoms; therefore, the *tweets by COVID long haulers* are critical to separate out of the overall conversation, as they provide direct insight into the concerns and experiences of this community. Of interest, however, was the finding of “medical care” as a frame in both data sets. Although themes within the frame differed based on record type, overall undertones for the urgency to diagnose and treat long COVID-19 appropriately as a medical condition existed, further acknowledging the clinical significance of this study. In addition, research methods that support higher levels of participant/patient engagement as well as study designs that are participant/patient-centered have been found to yield more successful study outcomes [39-41]. This study provides findings that may help to generate future research questions in a participant/patient-centered way as the discourse provided from Twitter indicates frames of interest. When it comes to those experiencing long COVID-19, Twitter users included in this study emphasized the need for support as well as describing their unresolved symptoms. These frames may be important topics for future research studies, placing a focus on patients’ immediate needs. Since COVID-19 is novel, and long COVID-19 is an emerging health crisis [16], the frames patients are interested in should have urgency.

Confirmation bias, the mechanism of seeking out and/or preferring information supporting prior beliefs [42,43], can offer an explanation into how both the framing and valence of tweets surrounding the topic of long COVID-19 develop and evolve. Within the employed frames, a trend of needing, seeking, or wanting to provide support can be identified across the two delineated conversations in this analysis. The frames “support” and “building a community” were predominant for *tweets about long COVID-19* and *tweets by COVID long haulers*, respectively. The suggestion of support and community building within each frame included various aspects of championing long COVID-19, containing financial, emotional, and informational context. Importantly, for the patient population, Twitter may be acting as a space for COVID-19 long haulers to validate their experiences and create a sense of community. The suggestion that SNSs give a lexicon by which users explain what they are going through emphasizes the bottom-up emergent conceptualization of this health issue and the connection with others that support their beliefs. Moreover, supportively framed tweets were most often of positive valence. However, the 29 records in *tweets about long COVID-19* exposing mistrust and conspiracy concerning long COVID-19 reflects a broader conversation about the politics of crisis and relates to confirmation bias [44]. The pandemic itself has been highly politicized, and political ideology has heavily influenced the way people conceptualized the pandemic and followed regulations such as social distancing, even more so than demographics such as age and income [45]. Our finding may be explained in part by the fact that sharing intention of health messaging on SNSs increases if it is appropriately leveraging the users’ confirmation bias, regardless of content valence [46]. In addition, Twitter users tend to reuse hashtags that were used very recently by their own and/or by their Twitter followees, indicating the temporal influence of confirmation bias [47]. Therefore, evaluating the influence of social hashtags exposure by investigating retweet or mention networks in Twitter has been identified as a future direction to study confirmation bias [47], and using SNA can assist in better understanding these phenomena.

The transmission and adoption of long COVID-19 discourse on Twitter appear to be highly decentralized, fragmented, and loosely connected. These findings are similar to a recent study that also used Netlytic to understand public discourse on Twitter around the COVID-19 pandemic [31]. This network type is not entirely surprising due to the nature of Twitter, as it was not designed to support the development of online communities but rather was imagined as a tool to share updates with others [30]. Moreover, online conversations are typically dominated by the few who are willing to post, resulting in predominantly parasocial or one-sided interactions, and research suggests that individuals are less likely to participate in conversations on sensitive topics because of the possible associated stigma [48]. Stigma and discrimination have been associated with those that have become ill with COVID-19 [49,50], which may in turn be impacting the network typology. Although previous literature has reported that SNSs offer a space for patients with newly described or rare heath concerns to find and connect with others similar to them [51,52], it appears that users in this study are participating in “lurking” behavior (ie, silently observing tweets and do not communicate) [53]. Knowing and understanding how this community of users typical behaves online can provide guidance for those attempting to disseminate health information and messaging on long COVID-19.

Overall, the network typology presented here (decentralized, fragmented, and loosely connected) has been shown to hinder the successful dissemination of risk communication by public health officials and health agencies across the network [31]. This is an important consideration due to the novelty of long COVID-19, and the way in which COVID-19 long haulers appear to be utilizing SNSs and the digital environment to find support and connect to others with similar experiences. However, it is important to examine the network properties individually and interpret how measures could potentially be leveraged within networks. In *tweets about long COVID-19*, the diameter property was larger than that in *tweets by COVID-19 long haulers*. A larger diameter can suggest that that information originating inside the core nodes also reaches people and communities far outside its core group of participants, which could be positive for spreading health messaging. Within both data sets, it appears that users are broadcasting information and not having conversations. This again may be beneficial for informational aspects of conversations about long COVID-19, such as resources and research findings and funding, all of which appeared as themes in this study. Moreover, there might be individual clusters in the network that are higher in density and reciprocity. Within these theoretical, closely knit clusters or niches, back-and-forth conversation would be occurring and thus satisfying the ideals of support conveyed in the data set. Future research using a microlevel SNA would be needed to explore this potential phenomenon.

### Limitations

Several limitations of this study need to be acknowledged. First, qualitative data have the potential for researcher bias; however, using Netlytic to complement the manual coding provided a more objective analytic tool as researcher bias, coder reliability, and subjectivity were diminished. Second, the data analysis and interpretation of social media were limited to Twitter; therefore, examining a wider array of user-generated comments on a variety of websites (eg, newspaper websites, discussion forums) and other SNSs would provide additional context. Although a contextually purposeful window of data collection [54,55] was chosen by the authors, future studies could include a more longitudinal design, thus following the trend of hashtags over time. In addition, including a geographic analysis might be of interest as the COVID-19 pandemic and subsequent long COVID-19 have had a global impact. Lastly, due to Twitter’s API restrictions, Netlytic limits data collection to 1000 tweets every 15 minutes, based on the data specifications given by the research. In other words, the tweets analyzed do not represent all of the tweets that were posted and do not include tweets from people who wrote about long COVID-19 but did not use the #longCOVID and #longhaulers hashtags. However, this study has important strengths, including frame overlap with human coding, as this allowed for a more robust interpretation of the data. Additionally, involving patients in clinical practice guidelines or the development of research questions typically is limited to a few representatives due to budgetary and logistical constraints [56,57]. In utilizing Twitter conversations, this study has proactively engaged a wider group of patients.

### Conclusion

Our results suggest that a popular SNS such as Twitter can effectively serve as a platform for the sharing of information and personal experiences related to long COVID-19. Records about long COVID-19 and records posted by users experiencing long COVID-19 exposed a variety of perspectives, including calls for research, political opinions, and the sharing of personal struggles. The findings indicated that tweeting about long COVID-19 is more commonly for informative purposes than for starting conversation. Future research may look at discourse occurring on SNSs that are aimed at facilitating group conversation, such as Facebook. Additionally, long COVID-19 research generally should seek to address the thoughts and experiences of the people affected by the disease to maximize impact.

## Abbreviations

API: application programming interface
SNA: social network analysis
SNS: social networking site
